# Development of goal-oriented behavior in self-learning robots

**DOI:** 10.1186/1471-2202-12-S1-P149

**Published:** 2011-07-18

**Authors:** Georg Martius, J Michael Herrmann

**Affiliations:** 1 Max Planck Institute for Mathematics in the Sciences, Inselstr. 22, 04103 Leipzig, Germany; 2 Bernstein Focus: Neurotechnology, Bunsenstr. 10, 37073 Göttingen, Germany; 3 University of Edinburgh, IPAB & ILSI, School of Informatics, 10 Crichton St, Edinburgh, EH8 9AB, UK

## 

The homeokinetic principle [1] describes a mechanism for the self-organisation of behaviour in early development. It implies a self-tuned balance between the sensitivity of motor actions with respect to sensory inputs and the predictability of the perceptual consequences of actions. The principle gives rise to a synaptic plasticity rule for artificial motor neurons, which has been shown to generate coherent and coordinated movements in autonomous robots [2] and which can be interpreted as a model of early behavioural learning. Learning in this sense consists in the construction of a behavioural manifold which must, however, remain modifiable in order to incorporated goal-related information or rewards in the course of further development. Goal-related optimization for shaping rather than replacing on-going exploration is referred to as guided self-organisation and is the subject of the present paper.

We present three strategies for guided self-organization, namely using rewards, teaching signals or assumptions about the symmetries of the desired behaviour. The strategies are analysed for several different robots (a khepera-like robot, spherical robot, snake, and multi-segment chain robot) in a physically realistic simulation [2].

## Guidance by reward

An online reward signal can act as a global modulation of the learning speed. The reward signal can now be used to bias the exploratory behaviour. In a spherical robot fast locomotion by rewarding high forward velocity is achieved as well as curved driving and spinning when rewarding high rotational velocity. In a more challenging example, a snake with 20 degrees of freedom develops various crawling behaviours when rewarded for high velocity.

## Guidance by teaching

If target values for motors or sensor are given, a natural gradient approach is found to be optimal also when embedded in a dynamical system. In experiments with a spherical robot, revolving behaviour about different axes is achieved by a perceptual teaching scheme based on a single sensor at a time.

## Guidance by cross-motor teaching

Internal teaching signals are generated by exploiting symmetries in the motor patterns of a desired behaviour, which are realised as mutual teaching between the motor units. This self-supervised scheme induced soft constraints that reduce the effective dimension of the dynamics and thus guide the self-organisation process into a sub-space of the control problem. The effectiveness of the method is demonstrated using a multi-segment chain robot which develops locomotion within a very short time. The direction of locomotion can be inverted by changing the mutual teaching scheme.

Constraining the process of behavioural self-organisation by a given or evolutionarily acquired objective leads to an exploration of behaviours within a lower dimensional manifold. This manifold characterises all behaviours that are compatible with the objective and that can be represented by the internal model of the robot. It is also possible that the external goal does not allow for the calculation of a gradient. Here the exploration produces cases that can be compared from the point of view of the robot such that learning becomes possible also under very general conditions. In the context of development the influence of learning by self-organisation and by reward mechanisms may vary. Although in the early stages the pure self-organisation of sensorimotor loops can be expected to follow mainly intrinsic principles, later stages will see a combination of different learning mechanisms as described here in an exemplary case. We find that the maintenance of criticality that is essential in the homeokinetic approach is not abandoned with goal-oriented learning as rather weak effects of the objective are most efficient. It can, furthermore, be predicted that an early exploratory phase which is not subject to directed learning increases the efficiency with which later the objective is met.

## References

[B1] Der R Self-organized acquisition of situated behavior Theory in Biosciences 2001 120 179 187

[B2] Martius G Ressources on homeokinesis 2011 http://robot.informatik.uni-leipzig.de21516058

